# Editorial: Neuromodulation in COVID-19: From basic research to clinical applications

**DOI:** 10.3389/fphys.2023.1148819

**Published:** 2023-02-16

**Authors:** Eugenijus Kaniusas, Marat Fudim, Christopher J. Czura, Fivos Panetsos

**Affiliations:** ^1^ Instutute of Biomedical Electronics, Faculty of Electrical Engineering and Information Technology, Vienna University of Technology (TU Wien), Vienna, Austria; ^2^ Division of Cardiology, Duke University Medical Center, Durham, NC, United States; ^3^ Duke Clinical Research Institute, Duke University, Durham, NC, United States; ^4^ Convergent Medical Technologies, Inc, New York, NY, United States; ^5^ Neurocomputing and Neurorobotics Research Group, Universidad Complutense de Madrid, Madrid, Spain; ^6^ Institute for Health Research (IdISSC), San Carlos Clinical Hospital, Madrid, Spain; ^7^ Silk Biomed SL, Madrid, Spain

**Keywords:** neuromodulation, COVID-19, vagus nerve, inflammation, autonomic function, dysautonomia

Neuromodulation with electrical nerve stimulation has come progressively into focus as a treatment option for various chronic diseases to change end-organ function through systemic effects on the human body (Chen et al., 2020; Cirillo et al., 2022; Kaniusas et al., 2019a). Stimulation affects multiple immunological, physiological, psychometric, and biochemical functions (Tracey, 2007; Krzyzaniak et al., 2011; Mercante et al., 2018). The brain chemistry, nociceptive processing, inflammation, and autonomic function are modulated by neurostimulation for different therapeutic purposes (Li et al., 2015; Babygirija et al., 2017; Kaniusas et al., 2019a). Even though neuromodulation therapy is generally considered safe with only mild and transient adverse effects (Redgrave et al., 2018; Kim et al., 2022), the mechanistic understanding of neuromodulation is still incomplete (Yap et al., 2020). While methodological standardization of the therapy is in progress (Farmer et al., 2021), personalization of neuromodulation is still insufficient (Yu et al., 2022; Kaniusas et al., 2019b) for a successful translation of neuromodulation into mainstream clinical practice (Goggins et al., 2022).

The COVID-19 pandemic may lead to severe respiratory distress, systemic inflammation, cardiovascular damage, and imbalance of the autonomic function, involving acute and chronic damages (Huang et al., 2020; Zheng et al., 2020; Tay et al., 2020). The COVID-19 infection affects several organ systems with different timelines, which suggests the use of electrical neuromodulatory approaches—with their aforementioned systemic regulatory effects—as a very promising and prospective treatment option, during and even before and after COVID-19 (Bonaz et al., 2020; Guo et al., 2021; Badran et al., 2022; Rangon and Niezgoda, 2022; Baptista et al., 2020; Fudim et al., 2020; Kaniusas et al., 2020).

The present Research Topic entitled “Neuromodulation in COVID-19: from basic research to clinical applications” sets out to shed light on a better understanding of the use of neuromodulatory approaches as potential treatment options for Covid-19-related diseases which continue to challenge our society.

For this proposed transdisciplinary exchange from basic science to engineering Research Topic and to clinical applications, we received a number of manuscript proposals, six of which were ultimately accepted for publication. While mechanistic Research Topic on the cellular level are covered by contributions Petrone et al. and Jankauskaite et al., clinical research in COVID-19 patients is reported by Tornero et al. and Seitz et al., all complemented by two review papers by Czura et al. and Linnhoff et al. for clinical settings.

Petrone et al. investigated in her research paper antibody and cellular responses to the ancestral strain and the Delta variant of Corona virus in vaccinated patients with an autoimmune disorder, namely, multiple sclerosis (MS). While cellular responses remained largely intact in MS patients, the magnitude of the antibody responses was reduced in patients treated with specific MS drugs (ocrelizumab or fingolimod) in comparison with subjects without MS. Authors substantiated the mechanistic action of the specific drugs on the vaccine-induced immune response to Corona viral variants in the real-life scenario of MS, and, in the broader sense, of other autoimmune disorders.

Jankauskaite et al. presented a hypothesis paper on the potential use of the vagus nerve stimulation (VNS) in Covid-induced lung injuries, addressing a highly susceptible pediatric population. This population is unfortunately underrepresented in the scientific literature, even though under risk and with distinct immunity and anatomy. The authors discussed the potential benefit of stimulation through inhibition of the damage-associated high-mobility group box protein (HMGB1). This protein is a predictive and initiating factor for inflammatory respiratory-system related diseases in COVID-19. The paper covered not only the cellular biophysics of VNS but also methodological issues and even potential risks of VNS in great depth, as applicable to the early development stage of pediatric population.

Tornero et al. conducted a prospective clinical study on the efficacy and safety of the non-invasive cervical VNS for the treatment of respiratory symptoms and over-inflammation in COVID-19. Patients were hospitalized for COVID-19 without the need for mechanical ventilation. While anti-inflammatory effects [e.g., on C-reactive protein (CRP)] of VNS were proven within 1 week, as well as safety of the VNS procedure, significant effects on the respiratory outcomes (e.g., oxygen saturation) were not observed. Authors argued that since the reduced pro-inflammatory markers are indicative of diminished risks of poor outcomes in COVID-19 (e.g., respiratory failure) as well as of cardiovascular complications (e.g., stroke), further studies of the cervical VNS are warranted.

Seitz et al. investigated the efficacy and safety of the minimally-invasive auricular VNS for the treatment of over-inflammation in COVID-19 within a prospective clinical study. In an instructive contrast to Tornero et al., only severe COVID-19 patients with the need for mechanical ventilation were included. Within 1 week, the auricular VNS reduced not only pro-inflammatory markers (e.g., CRP) but also increased favorably anti-inflammatory markers [e.g., interleukin 10 (IL-10)]; without any stimulation-related adverse events. Authors suggested the auricular VNS as a promising option for additional treatment in severe COVID-19 patients.

Czura et al. conducted a review on neuromodulation strategies to reduce over-inflammation and avoid respiratory complications in COVID-19 patients. The role of the nervous system (incl. vagus and sacral nerves) was systemically discussed for inflammation and respiration, followed by (pre)clinical rationale in using transcutaneous cervical and auricular VNS, electromagnetic transcranial, and focused ultrasound stimulations in Covid-19-related disorders. These neuromodulatory approaches could potentially be used also in systemic disorders triggered by other pathogens. The authors formed the International Consortium on Neuromodulation for COVID-19, which developed recommendations in neuromodulation studies in COVID-19.

Linnhoff et al. reviewed the potential of non-invasive brain stimulation technologies for the treatment of (cognitive) fatigue, the most common and debilitating symptom of Long-Covid. Neuromodulation methods such as transcranial direct and alternating current stimulation, as well as transcutaneous VNS were revised in view of their ability to modulate fatigue-related maladaptive neuronal circuitry; namely, dysregulated immune system, frontoparietal hypometabolism, and reduced cerebral blood flow.

Selected publications show that neuromodulation with its systemic effects on the human body has balancing modulatory effects on the systemically-derailed state in COVID-19 patients. Given the low risk profile of neuromodulation in view of severe complications in COVID-19, we conclude that neuromodulation can act as a promising and prospective treatment option in COVID-19.

Finally, we would like to thank the reviewers for their valuable comments and suggestions. Without their help, the publication of this Research Topic would not have been possible. We hope that the readers will find this collection of papers useful.
